# Identification and Characterization of Pheromone Receptors and Interplay between Receptors and Pheromone Binding Proteins in the Diamondback Moth, *Plutella xyllostella*

**DOI:** 10.1371/journal.pone.0062098

**Published:** 2013-04-23

**Authors:** Mengjing Sun, Yang Liu, William B. Walker, Chengcheng Liu, Kejian Lin, Shaohua Gu, Yongjun Zhang, Jingjiang Zhou, Guirong Wang

**Affiliations:** 1 State Key Laboratory for Biology of Plant Diseases and Insect Pests, Institute of Plant Protection, Chinese Academy of Agricultural Sciences, Beijing, China; 2 Swedish University of Agricultural Sciences, Department of Plant Protection Biology, Chemical Ecology Research Group, Alnarp, Sweden; 3 Department of Biological Chemistry, Rothamsted Research, Harpenden, United Kingdom; Plant and Food Research, New Zealand

## Abstract

Moths depend on olfactory cues such as sex pheromones to find and recognize mating partners. Pheromone receptors (PRs) and Pheromone binding proteins (PBPs) are thought to be associated with olfactory signal transduction of pheromonal compounds in peripheral olfactory reception. Here six candidate pheromone receptor genes in the diamondback moth, *Plutella xyllostella* were identified and cloned. All of the six candidate PR genes display male-biased expression, which is a typical characteristic of pheromone receptors. In the *Xenopus*-based functional study and *in situ* hybridization, PxylOR4 is defined as another pheromone receptor in addition to the previously characterized PxylOR1. In the study of interaction between PRs and PBPs, PxylPBPs could increase the sensitivity of the complex expressing oocyte cells to the ligand pheromone component while decreasing the sensitivity to pheromone analogs. We deduce that activating pheromone receptors in olfactory receptor neurons requires some role of PBPs to pheromone/PBP complex. If the chemical signal is not the pheromone component, but instead, a pheromone analog with a similar structure, the complex would have a decreased ability to activate downstream pheromone receptors.

## Introduction

Olfaction plays an indispensable role in mediating critical insect behaviors such as food selection, predator and noxious agent avoidance, and appropriate mating-partner choice [1], [2]. Chemosensory systems, such as taste and smell, involve a complex process from the peripheral transduction of the chemical signal through olfactory neurons to electrical signal processing in central nervous system [3]. In the field of insect olfactory research, moth pheromone communication is a valuable model system for studying the fundamental aspects of animal sensory perception at the molecular level, as the detection of female-released sex pheromone by male antennae is extremely sensitive and specific [4], [5]. In moths, the mating partner selection is mostly dependent on the sensitive identification of female-released pheromones by specialized sensory neurons in trichoid sensilla on male antennae [6].

Major findings of the molecular components of insect chemosensory systems over the past decade have improved our understanding of pheromone identification by male moths [7], [8], [9], [10], [11]. An important early contribution in this area has been thought of as the first identification and characterization of pheromone receptor (PR) in the silkworm moth *Bombyx mori* [12], [13]. The pheromone receptor BmorOR1 was identified through the following evidence: a) BmorOR1 encodes a 430-aa protein with seven putative trans-membrane domains that are characteristic to all odorant receptors; b) BmorOR1 is a male-antennae specific OR in RT-PCR experiments; this is a typical characteristic of pheromone receptors; c) BmorOR1 mRNA was localized to the specific cells in the male antennae in the region carrying chemosensory hairs, and PBP mRNA was expressed in supporting cells that surround BmorOR1; d) two-electrode voltage clamp recordings of *Xenopus* oocytes provide evidence that BmOR1 functions as a bombykol receptor in a heterologous cell system; e) ectopic expression of BmorOR1 in female antennae conferred responsiveness to bombykol indicating that BmorOR1 functions as a highly specific receptor for bombykol in the silkmoth antennae. Since the BmOR1 was identified and functionally characterized, other moth pheromone receptors have been studied through some or all of the above experiment methodologies. These receptors are primarily identified by genomic sequence [12], [14], transcriptomic sequence [15], [16], [17], [18] and homologous cloning [19], [20], [21].

In addition to pheromone receptors, there is another important class of proteins associated with olfactory mechanisms in peripheral olfactory reception. Soluble pheromone binding proteins (PBPs), are a subfamily of odorant binding proteins in the aqueous sensillar lymph, and are thought to facilitate transportation of hydrophobic sex pheromone components emitted by conspecific female across the sensillar lymph to the surface of olfactory receptor neurons [2], [22]. Previous studies have provided evidence that PBPs could increase physiological sensitivity to pheromone ligands. When pheromones were solubilized by PBPs, threshold responses of PRs from *B. mori*, *Heliothis virescens*, and *Antheraea polyphemus*, heterologously expressed in HEK293 cells, were much more sensitive than when pheromones were solubilized by DMSO [19], [23], [24]. Similar results were obtained by expressing BmorOR1 in the “empty neuron” of *Drosophila melanogaster* [25], [26]. PBPs may also affect the PR’s physiological specificity to sex phoromone. A PBP added to an *in vitro* assay altered the specificity of a moth pheromone receptor, making its response more specific [23]. There is yet another theory that PBP/pheromone complexes are not necessary for activation of moth PRs, as receptor activity was dramatically reduced when the ligands were solubilized by BmorPBP1 when heterologously expressed in *Xenopus* oocytes [27]. Until now, different experimental systems show various results, so we still cannot accurately proclaim how PBPs interact with pheromones and PRs.

The diamondback moth, *Plutella xylostella* (Lepidoptera: Plutellidae), is an economically important vegetable pest in the world. Many cruciferous crop plants are damaged much by the moths’ invasions every year. Its pheromonal system has been broadly studied in recent decades, as a good target for mating disruption. The main pheromone components of diamondback moth, which have been extracted from the female moths’ abdomens, are (Z)-11-hexadecenal [Z11-16:Ald] and (Z)-11-hexadecenyl acetate [Z11-16:Ac] [28]. (Z)-11-Hexadecenol [Z11-16:OH] was subsequently identified as a pheromone component, which could increase the efficiency of moth attraction in the field with low concentrations [29], [30]. The most common sex attractant is the mixture of the above two or three components in different ratios [31], [32]. (Z)-9-tetradecenyl acetate [Z9-14:Ac] is also thought to be an additional pheromone component because it could attract significantly more male moths when added into a mixture of the previous three components at trace quantities [33], [34]. Some olfaction-associated genes in the diamondback moth have been characterized in recent years. So far, four OR genes (PxOR1, PxOR3, PxOR4, PxOR83b) have been cloned by way of homologous cloning [20]. PxOR83b is the co-receptor, which is called Orco or OR2 in Lepidoptera. PxOR1 has been identified as a pheromone receptor and activated by Z11-16: Ald in *Xenopus* oocytes [20].

In this study, six candidate pheromone receptors (including PxOR1, PxOR3 and PxOR4) were filtered and cloned according to transcriptomic sequence information. Among the cloned genes, we identified receptors of sex pheromones by using *Xenopus* oocytes and two-electrode voltage clamp recording. Three identified PBPs [35] were added in to further study the influence of PBPs on PRs, which could help to advance research on olfactory mechanisms in the moth and lay the foundation for developing novel strategies to control the pest.

## Materials and Methods

### Materials

*Xenopus laevis* frogs were purchased from Nasco. The care and use of *X. laevis* frogs in this study were approved by the Institute of Plant Protection, Chinese Academy of Agricultural Sciences Animal Research Committee and meet the guidelines of the National Institues of Health. Moths has been reared on Chinese cabbage in the laboratory in the Institute of Plant Protection, Chinese Academy of Agricultural Sciences, at 27±1°C, 16∶8 h light/dark photoperiod and 65±5% relative humidity.

### RNA Extraction and cDNA Synthesis

Antennae, heads (without antennae), proboscises, labial palps, genitals, thoraxes, abdomens, legs and wings of adult were collected from 1 to 3 day-old moths and stored at −70°C before use. Total RNA was extracted from antennae and other organs described above using the Trizol Reagent (Invitrogen, Carlsbad, CA) following the manufacturer’s instructions and then quantitated using Nanodrop (Inc., Wilmington, DE). The first-strand cDNA was synthesized from 2 µg of RNA with the Revert Aid First Strand cDNA Synthesis Kit (Fermentas, Glen Burnie, MD) following the manufacturer’s protocol.

### PCR Amplification

According to transcriptomic sequence data, six candidate pheromone receptors were identified and named as PxylOR1 and 3–7. The protein encoding, open reading frames (ORFs) of these genes were cloned using specific primers. The sequences of all primers are listed in Table 1.

**Table 1:** Primers for experiments.

Primer name	Sequence (5′–3′)
Specific primers for cloning	
PxylOR1-F	TACTCA*GCGGCCGC*gccaccATGCGTGTATTCTTCTTAACAG (*Not*I)
PxylOR1-R	TACTCA*GTCGAC*CTACTTTGAACGCAAAAATG (*Sal*I)
PxylOR3-F	TACTCA*GCGGCCGC*gccaccATGCCAGCAGGGGCAGTTTAC (*Not*I)
PxylOR3-R	TACTCA*GTCGAC*TTACTCGGCCCTAATCGATTTCAG (*Sal*I)
PxylOR4 -F	TACTCA*GCGGCCGC*gccaccATGAAACCTGGAGCTCTAAGCC (*Not*I)
PxylOR4-R	TACTCA*GTCGAC*TTATTCGCTGATTGATTTCAGTAAAG (*Sal*I)
PxylOR5 -F	TACTCA*GCGGCCGC*gccaccATGTCGAGGAAAGCAGGAGC (*Not*I)
PxylOR5-R	TACTCA*GTCGAC*TTATTCGCTTATCGATTTTAACAAAG (*Sal*I)
PxylOR6-F	TACTCA*GCGGCCGC*gccaccATGATACAAACAGGGGAGAGAAG (*Not*I)
PxylOR6-R	TACTCA*GTCGAC*TCAATCGTTTATTGATTGTAAAATC (*Sal*I)
PxylOR7 -F	TACTCA*GCGGCCGC*gccaccATGAACGAAAAGTATTTGGATCTGA (*Not*I)
PxylOR7-R	TACTCA*GTCGAC*TCATCCTTCATCGACTGTCACTAA (*Sal*I)
Primers for RT-PCR	
PxylOR1-RF	ATCCCCTTCATCGTCATCTACC
PxylOR1-RR	GCTGACCTGGTGGAACGAGTAG
PxylOR3-RF	GCTGAGATTTCTGCGTATTGGG
PxylOR3-RR	ACGCAGATGCTACACGCAGTTAT
PxylOR4 -RF	GGTTACCTTAGTGCGGTCATTGTTAC
PxylOR4-RR	GAAGTGGTCGTAGCAGTGGAAGC
PxylOR5 -RF	TGTTATCACAAGCACAAGGGAA
PxylOR5-RR	ATTCATCGTCGTAGATATGTAGAAGTG
PxylOR6-RF	ATGCAGATGACGCTGATGGTA
PxylOR6-RR	TCAATGGAGCAAACTGACACG
PxylOR7 -RF	TTGTGGCGTCACTCACTGTTC
PxylOR7-RR	TTGTAACTGTTGAATATCGGTATTCC
PxylActin-RF	GGAGTGATGGTCGGTATGGG
PxylActin-RR	TGGGTCATCTTTTCACGGTTG
Primers for Real-time PCR	
PxylOR1-QF	CATCCCCTTCATCGTCATCTACC
PxylOR1-QR	GCGGTAGTAAAAGCCTGGTCG
PxylOR3-QF	TTGCCACATTTTGAAGAATACAGAA
PxylOR3-QR	CAATACGCAGAAATCTCAGCCTC
PxylOR4 -QF	GTTACCTTAGTGCGGTCATTGTTAC
PxylOR4-QR	CCTCATATTTGCCTTTAGCCTTG
PxylOR5 -QF	CCTCTGCTCATCCGATACCAC
PxylOR5-QR	ACATCTCATCGTTAAATTTCCACA
PxylOR6-QF	CTGCTTTCTTACTTTGGCTACTGG
PxylOR6-QR	ACTTTTTCATCGTACTCTCCCTTG
PxylOR7 -QF	TATGGCAGCCAAATGTAGTCTAAC
PxylOR7-QR	GCAATAGTGCGAAATCTTGTCTAC
PxylActin-RF	GCCGTCTTCCCGTCCAT
PxylActin-RR	GATACCTCTCTTGCTCTGGGC

Note. The restriction enzyme of each primer is marked in parenthesis behind the sequence, and the cutting sites are inclined.Kozak sequence is lower case.

The PCR reaction mixture contained 17 µL ddH_2_O, 2.5 µL 10×ExTaq Buffer, 2 µL dNTPs (2.5 mM), 1 µL cDNA, 1 µL forward primer (10 µM), 1 µL reverse primer (10 µM), and 0.5 µL ExTaq DNA polymerase (Takara, Dalian, Liaoning, China). PCR reaction conditions: 94°C for 3 min; 35 cycles of 94°C for 30 s, 52–55°C for 45 s, and 72°C for 90 s; 72°C for 10 min.

### Sequencing and Phylogenetic Analysis

PCR products were separated by electrophoresis on 1% agarose gels in 1×TAE buffer. The specific fragments were cut and purified by DNA purification kit (TianGen, Beijing, China) following the manufacture’s protocol. The purified products were ligated with pGM-T Vector (TianGen) and then transformed into Top10 *E. coli* competent cells (TianGen). Positive clones were selected and sequenced (BGI, Beijing, China).

Sequences of odorant receptors including pheromone receptors in *B. mori*, *H. virescens*, *Helicoverpa armigera and Manduca sexta* from GenBank were used for phylogenetic analysis. A phylogenetic tree was reconstructed with the neighbor-joining method in MEGA 5.0 software (The Biodesign Institute, Center for Evolutionary Functional Genomics, Tempe, AZ). Branch support was assessed by bootstrap analysis based with 1,000 replications.

### Expression Profiles of Six Candidate Pheromone Receptors

Tissue expression patterns of six candidate pheromone receptors were assessed by RT-PCR with the cDNA templates from antennae and other tissues of male and female moths. Specific primers were designed according to the cDNA sequences. Integrity of the cDNA templates was tested with a pair of control primers from the coding region of the *P. xylostella* actin gene (GenBank AB282645). The sequences of all primers are listed in Table 1. PCR began at 95°C for 3 min, then 35 cycles at 94 °C for 30 s, 54°C for 30 s, and 72°C for 30 s, with a final 10 min’s incubation at 72°C. PCR products were analyzed on 2.0% agarose gels.

Quantitative real-time PCR was performed in an iCycler iQ2 Real-Time PCR Detection System (Bio-Rad, Hercules, CA) with SYBR green dye bound to double-strand DNA at the end of each elongation cycle (Promega, Madison, Wisconsin). Each pair of PCR primers was designed to span a cDNA exon-exon border to avoid amplification of potential traces of genomic DNA [36]. The primers of PxylOR1 and 3–7 amplified 120 bp, 110 bp, 112 bp, 100 bp, 135 bp and 151 bp fragments from cDNA template, respectively. As an endogenous control to normalize the results of a variable target gene and to correct for sample-to-sample variation, the *P. xyllostella* actin gene was used, for which the expected PCR product of was 108 bp. The sequences of all primers are listed in Table 1.

qRT-PCR was conducted in 20 µL reactions that contained 10 µL of 2×SYBR Green PCR Master Mix, 1 µl of each primer (10 µM), 1 µL of sample cDNA and 7 µL sterilized ultrapure H_2_O. The cycling parameters were: 95°C for 3 min; 40 cycles of 95°C for 10 s, 55°C for 30 s. To confirm reproducibility, test samples and the endogenous control were assayed in triplicate with three biological samples. Gene expression levels in male antennae and female antennae were calculated using the comparative 2^–ΔΔCt^ method [37]. △Ct.X = Ave.ORXCt-Ave.actinCt, △△Ct = △Ct.X-△Ct.female.PxylOR3 (X is male or female of other genes). Sexual differences of qPCR data was analyzed by one-way analysis of variance (ANOVA).

### Receptor Expression in Xenopus Oocytes and Two-electrode, Voltage-clamp Electrophysiological Recordings

The entire coding region of each PxylOR was sub-cloned into the *Not*I/*Sal*I sites of pSP64T-Oligo vector (Invitrogen) (Kozak sequence added behind the cutting site in forward primer). cRNAs were synthesized from linearized vectors with mMESSAGE mMACHINE SP6 (Ambion).

The methods of later electrophysiological recordings were according to previously reported protocols [38], [39]. Mature healthy oocytes (stage V–VII) (Nasco, Salida, California) were treated with collagenase I(GIBCO, Carlsbad, CA) in washing buffer (96 mM NaCl, 2 mM KCl, 5 mM MgCl_2_, and 5 mM HEPES [pH = 7.6]) for about 1 h at room temperature. After being cultured overnight at 18°C, oocytes were microinjected with 27.6 ng PxylORs cRNA and 27.6 ng PxylOrco (PxylOR83b) cRNA. After injection, oocytes were incubated for 4–7 days at 18°C in 1X Ringer’s solution (96 mM NaCl, 2 mM KCl, 5 mM MgCl_2_, 0.8 mM CaCl_2_, and 5 mM HEPES [pH = 7.6]) supplemented with 5% dialysed horse serum, 50 mg/ml tetracycline, 100 mg/ml streptomycin and 550 mg/ml sodium pyruvate. Whole-cell currents were recorded from the injected *Xenopus* oocytes with a two-electrode voltage clamp. Odorant induced currents were recorded with an OC-725C oocyte clamp (Warner Instruments, Hamden, CT) at a holding potential of −80 mV. Data acquisition and analysis were carried out with Digidata 1440A and pCLAMP 10.2 software (Axon Instruments Inc., Union City, CA). Statistical comparison of responses of oocytes to the tested ligands was assessed using one-way analysis of variance (ANOVA) procedure in SPSS 17.0 (Statistical Product and Service Solutions, Chicago, Lllinois). Dose-response data were analysed by GraphPad Prism 5.0 (GraphPad Software Inc., San Diego, CA).

Tested pheromone components (Z11-16:Ald, Z11-16:Ac, Z11-16:OH and Z9-14:Ac) and analogs (Z9-16:Ald, 16:Ac and Z9,E12-14:Ac) were purchased from Bedoukian (Danbury, CT) (purity>95%); they were dissolved in dimethyl sulfoxide (DMSO) to 1 M Stock solutions and stored at −20°C. Before testing, the stock solution was diluted with 1 X Ringer’s buffer (96 mM NaCl, 2 mM KCl, 5 mM MgCl_2_, 0.8 mM CaCl_2_ and 5 mM HEPES [pH = 7.6]).

### *In situ* Hybridization

Male antennae of 1 to 3 day-old moths were embedded in Jung tissue-freezing medium (Leica, Nussloch, Germany) and stored at −70°C before use. Cryosections (7 µm) were thaw-mounted on anti-off slides using a cryostat (Leica CM1850; Leica Microsystems, Wetzlar, Germany) and air-dried at room temperature for about 30 min.

Digoxigenin (DIG)-labeled sense and antisense probes were generated from linearized recombinant pGM-T plasmids using the T7/SP6 RNA transcription system following the manufacturer’s protocol (Roche, Basel, Switzerland). The later preparation of synthesized riboprobes and the procedures of *in situ* hybridization were performed as described in previously reported protocols [40], [41].

Antennae were analyzed on an Olympus microscope (Olympus, Tokyo, Japan) equipped with Cellsens Dimension software. Images were not altered except for adjusting the brightness or contrast for uniform tone within a single picture.

### Recombinant protein expression and purification of pheromone binding proteins (PBPs)

The entire coding region without signal peptide sequence of each PxylPBP was sub-cloned into the *Eco*R I/*Xho*I sites of PET30a (+) vector (Novagen, Madison, WI). BL21 (DE3) *E.coli* competent cells (Tiangen) were transformed by heat shock and colonies were grown on LB kanamycin (25 mg/mL) agar plates. Single positive colony was first identified and then grown in 5 mL liquid LB with 100 µg/mL kanamycin overnight at 37°C. The culture was diluted to 1∶100 in fresh medium, and cultured for 2–3 h at 37°C until its OD_600_ reached 0.4–0.6. IPTG was added into the culture with a final concentration of 1 mM and then the culture was incubated at 27°C for 12 h. After that, the cells were collected with centrifugation (8,000 rpm, 5 min) and dissolved with 1× phosphate-buffered saline (PBS). The suspension was crushed by sonication, and then separated into supernatant app:ds: supernateand sediment by centrifugation (12,000 rpm, 10 min, 4°C). The supernatant was purified by HisTrap affinity columns (GE Healthcare Biosciences, Uppsala, Sweden) and then dialyzed overnight at 4°C. Once the protein was concentrated, the His-tag on the recombinant protein was cut off by recombinant enterokinase (rEK) (Novagen), and then the digested protein was purified by HisTrap affinity columns again. After dialyzed with 50 mM Tris-HCl (pH = 7.4) overnight at 4°C, the protein was concentrated at last. The purity was checked by SDS-PAGE, and the concentration was determined by calibration curve method.

The purified PBPs were dissolved in 1X Ringer’s solution to final concentration of 1 µM. To analyze the ability of the different PBPs to solubilize and transport specific pheromone components and to influence the functions of the defined pheromone receptor, identified ligands were dissolved in Ringer solution with PBPs processed as above. Electrophysiological recordings and result analysis were performed according to the protocols shown above.

## Results

### Sequence and Phylogenetic Analysis of Six Candidate Pheromone Receptors

**Figure 1:**
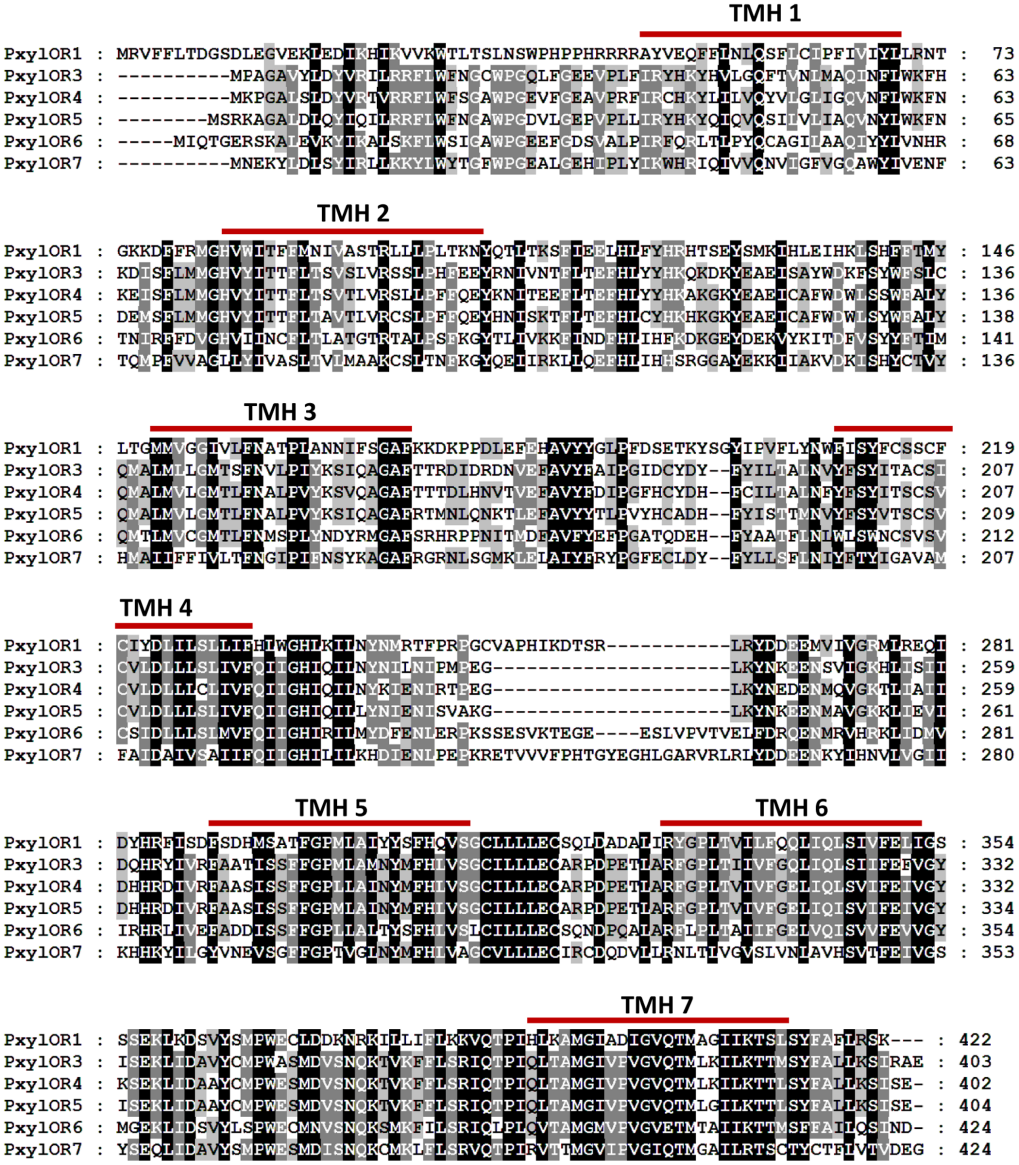
Sequence analysis of six candidate pheromone receptors. Seven putative trans-membrane domains are indicated with red bar and “TMH ‘n’” designation, where ‘n’ designates sequential order of the putative transmembrane domain. Trans-membrane domains were predicted by TMHMM Server v. 2.0 (http://www.cbs.dtu.dk/services/TMHMM/).

**Figure 2:**
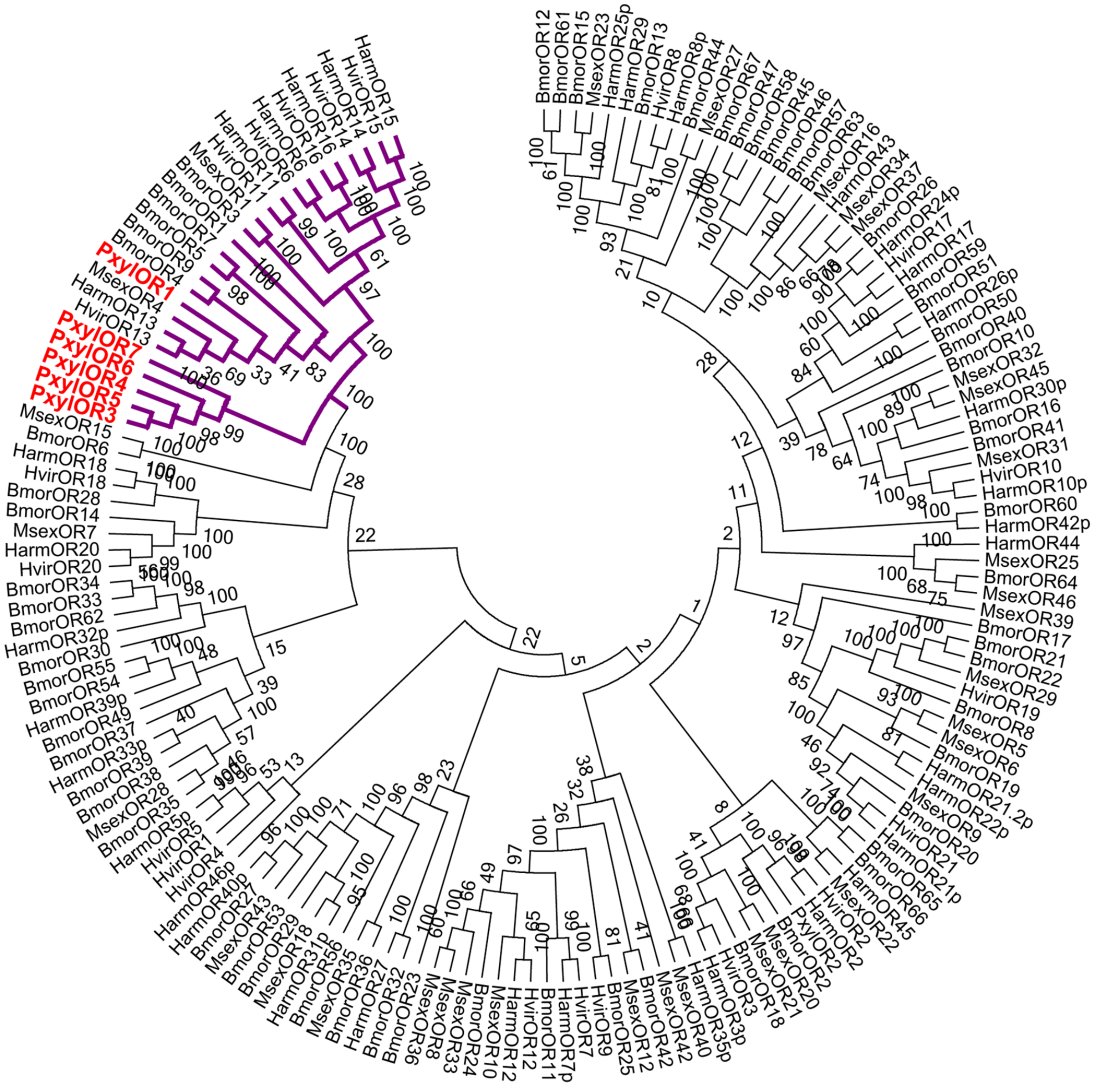
Phylogenetic analysis of six candidate pheromone receptors. The branch colored in purple represents the sub-grouping of pheromone receptors. GenBank accession numbers are listed in Material S1.

PxylOR1 and 3–7 (GenBank KC538876- KC538881) contain ORFs of 1269 bp, 1212 bp, 1209 bp, 1215 bp, 1275 bp and 1275 bp, respectively. All of them contain seven putative trans-membrane domains that are characteristic to all odorant receptors in insects (Figure 1) [42]. According to paired-sequence alignment results, PxylOR1, PxylOR3, and PxylOR4 are defined as PxOR1, PxOR3, PxOR4 as described previously (data are not shown) [20]. PxylOR5, PxylOR6 and PxylOR7 have been named sequentially. Phylogenetic analysis shows the six candidate pheromone receptors cluster together in the group of pheromone receptors (Figure 2). PxylOR1 is similar to MsexOR4 (GenBank ADM32897), HarmOR13 (GenBank ACS45304) and HvirOR13 (GenBank CAG38114) with identity values of 43.42%, 45.35% and 43.95%, respectively. While PxylOR3-7 group together in this phylogenetic tree indicating that the functional specialization of these receptors in *P. xyllostella* is very high.

### PxylOR1 and 3–7 are Expressed at Higher Levels in Male Antennae

**Figure 3:**
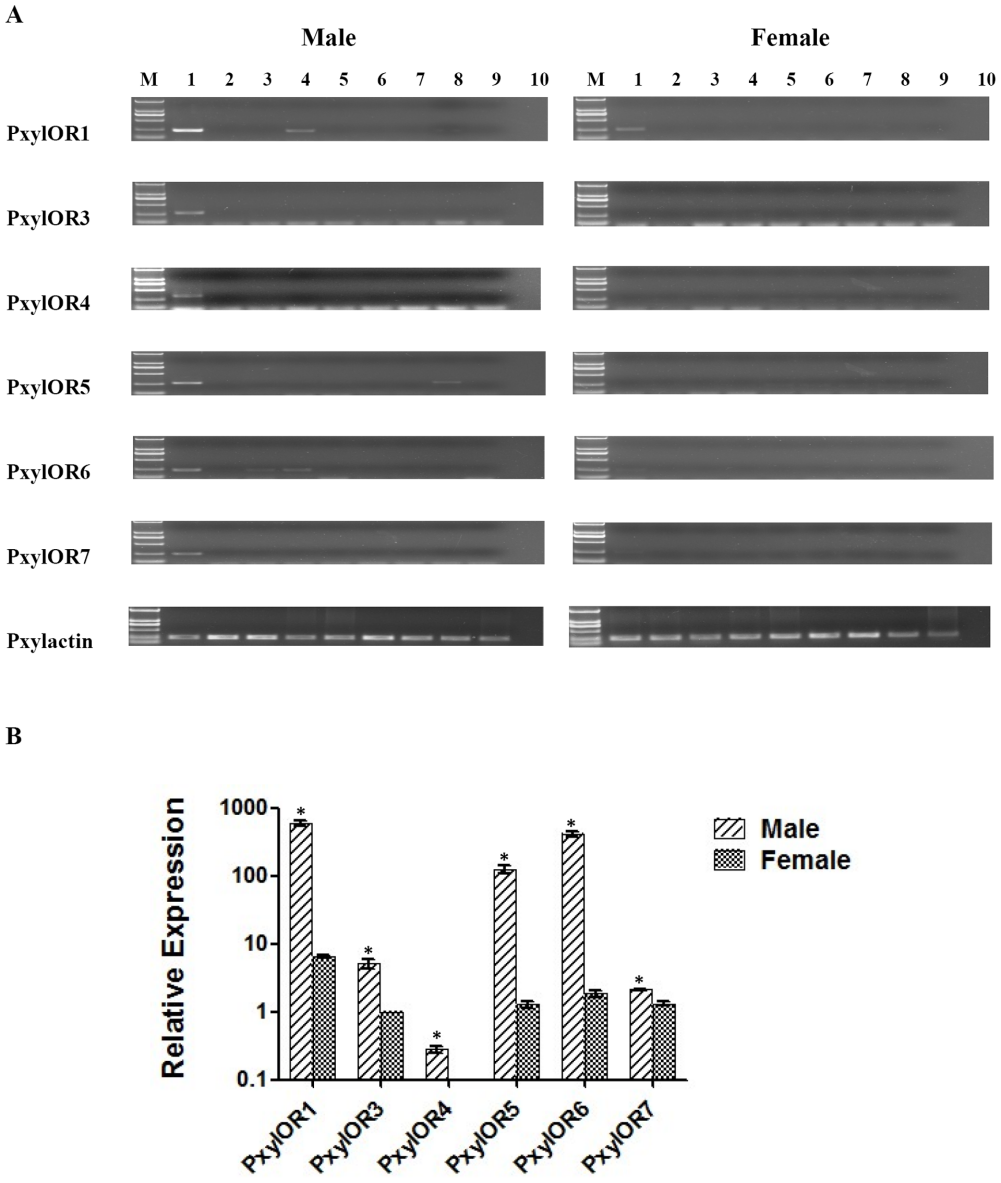
Tissue specificity and expression pattern of six candidate pheromone receptors amplified in male and female moth. (**A**) tissue specificity of PxylOR1 and 3–7; M is D2000 marker (Tiangen), 1 is antennae, 2 is heads (without antennae), 3 is proboscis, 4 is labial palps, 5 is genitals, 6 is throats, 7 is abdomens, 8 is legs and 9 is wings, 10 is negative control. (**B**) Relative expression quantity of six candidate pheromone receptors amplified in male and female antennae. The x-axis shows the candidate pheromone receptors in male and female moths, the Y-axis indicates relative expression quantity (mean+standard error of mean). The expression of female PxylOR3 is taken as the reference standard.

RT-PCR experiments were first performed to determine the tissue expression pattern of six candidate pheromone receptor genes (Figure 3A). For PxylOR3-5, PCR bands were only obtained with cDNA from male antennae. For PxylOR1, PxylOR6 and PxylOR7, PCR bands were obtained with cDNA from both male and female antennae, but with a striking difference of expression levels in two sexes, namely that a much stronger band was obtained with cDNA from male antennae. Additionally, PxylOR1 and PxylOR6 genes are also faintly expressed in maxillary palps in male moths. Occasionally for PxylOR5, a very faint band is also obtained with cDNA from male legs.

qRT-PCR was further performed to precisely measure the transcript level of six candidate pheromone receptor genes in male and female antennae. The results show that all of the six candidate genes display male-biased expression, which is typically characteristic of pheromone receptors (Figure 3B).

### Specific Responses of Different Sex Pheromone Receptors

Each of the six candidate pheromone receptors was co-expressed in *Xenopus* oocytes with the non-ligand binding functional co-receptor partner PxylOrco, and screened for responsiveness to a panel of pheromone components (Z11-16:Ald, Z11-16:Ac, Z11-16:OH and Z9-14:Ac) and analogs (Z9-16:Ald, 16:Ac and Z9, E12-14:Ac), at a 100 µM concentration. After activation stimulus was applied, oocytes were thoroughly washed until a steady baseline was reached.

**Figure 4:**
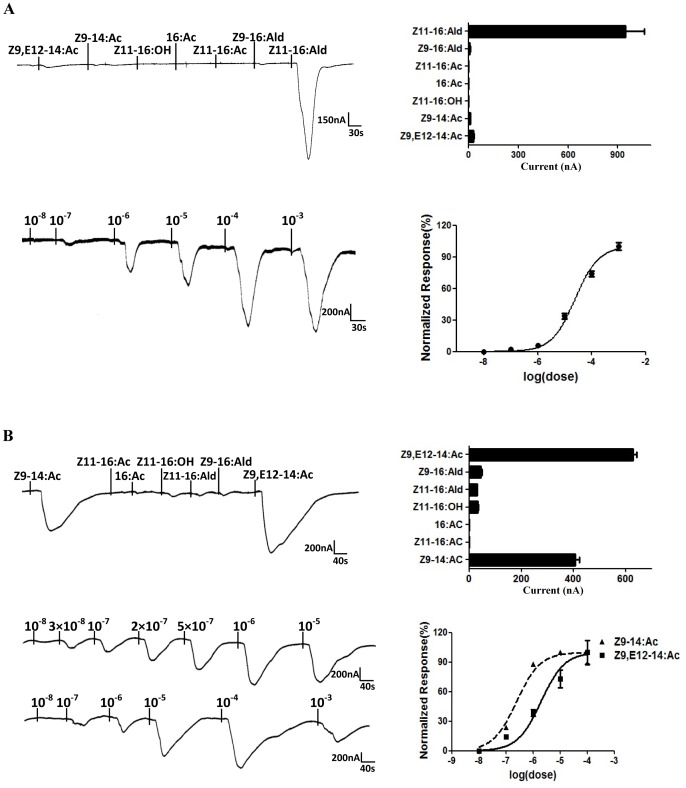
Responses of *Xenopus* oocytes with co-expressed PxylOR1/PxylOrco or PxylOR4/PxylOrco to stimulation with pheromone compounds. (**A**) (Upper left) Inward current responses of PxylOR1/PxylOrco *Xenopus* oocytes in response to 10^−4^ M of pheromone compounds and analogs. (Upper right) Response profile of PxylOR1/PxylOrco *Xenopus* oocytes. Error bars indicate SEM (*n = *6). (Lower left) PxylOR1/PxylOrco *Xenopus* oocytes stimulated with a range of *Z*11-16: Ald concentrations. (Lower right) Dose–response curve of PxylOR1/PxylOrco *Xenopus* oocytes to *Z*11-16:Ald. Responses are normalized by defining the maximal response as 100%. *Z*11-16: Ald EC50 = 2.39×10^−5^ (n = 5). Error bar indicates SEM. (**B**) (Upper left) Inward current responses of PxylOR4/PxylOrco *Xenopus* oocytes in response to 10^−4^ M of pheromone compounds and analogs. (Upper right) Response profile of PxylOR4/PxylOrco *Xenopus* oocytes. Error bars indicate SEM (*n = *7). (Lower left) PxylOR4/PxylOrco *Xenopus* oocytes stimulated with a range of pheromone Z9-14: Ac and analog Z9, E12-14: Ac concentrations, respectively. (Lower right) Dose–response curve of PxylOR4/PxylOrco *Xenopus* oocytes to Z9-14: Ac and Z9, E12-14: Ac. Responses are normalized by defining the maximal response as 100%. Z9-14: Ac EC50 = 2.43×10^−7^ (*n = *7) and Z9, E12-14: Ac EC50 = 1.94×10^−6^ (*n = *5). Error bar indicates SEM.

Only PxylOR1 and PxylOR4 were each successfully activated by one of the pheromone components. PxylOR1 is tuned to the major pheromone component, Z11-16:Ald, with a mean amplitude of about 1000 nA, which is consistent with a previous report (Figure 4A) [20]. PxylOR4 is tuned to a minor pheromone component, Z9-14: Ac and its analog, Z9, E12-14: Ac (Figure 4B). Although PxylOR4 responds to Z9, E12-14: Ac more strongly than Z9-14: Ac at a 100 µM concentration, the EC50 of Z9, E12-14: Ac is one order of magnitude higher than Z9-14: Ac, thus, PxylOR4 is tuned to pheromone component more sensitively.

### In situ Hybridization Demonstrates that PxylOR4 is a Pheromone Receptor

To further assess PxylOR4 as a pheromone receptor, *in situ* hybridization experiments of PxylOR1 and PxylOR4 were performed. As we know, PxylOR1 has been defined as a pheromone receptor. If *in situ* hybridization experiments show that the expression pattern of PxylOR4 is similar to that of PxylOR1, PxylOR4 could be further identified as a pheromone receptor.

**Figure 5:**
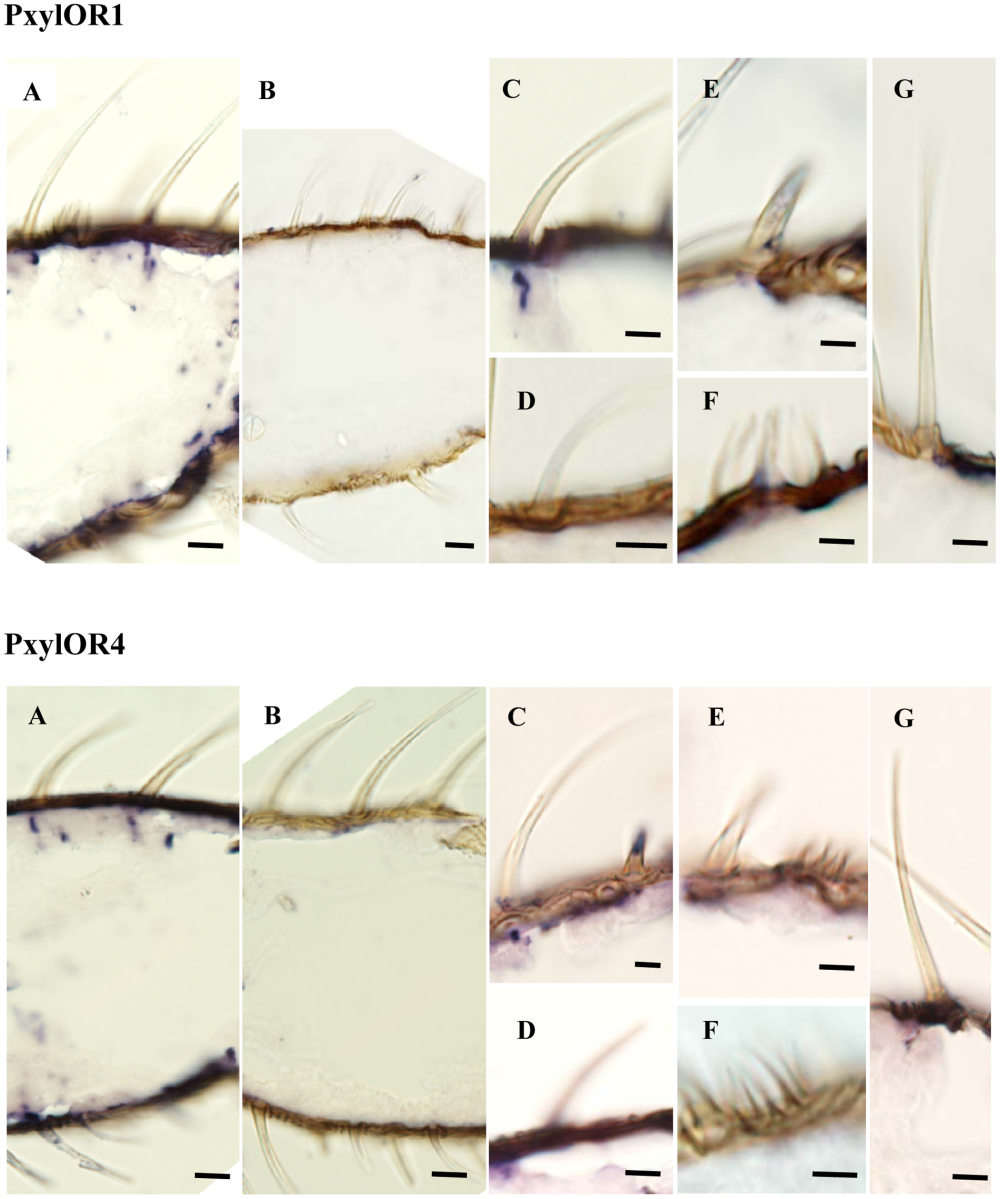
Expression of PxylOR1 and PxylOR4 genes in male antenna of *P. xylostella*. *In situ* hybridizations were performed with digoxigenin-labelled antisense RNA probes on longitudinal tissue sections of male antennae. Signals were visualized using an anti-DIG antibody. (**A**) Hybridization signals in one segment of the *P. xylostella* antenna are shown. (**B**) Negative control with a DIG-labeled sense probe. (**C**) Higher magnification of long trichoid sensilla with hybridization signals. (**D**) **to** (**G**) No hybridization signal was detected under short trichoid sensilla, basiconi sensilla, coeloconic sensilla and chaetica sensilla. Scale bars: 5 µm in A–B and 2 µm in C–G.

The *in situ* hybridization experiments were performed to visualize cells expressing PxylOR1 and PxylOR4 in cryosections of the proximal segments from male antenna (Figure 5A). PxylOR1 labeled cells are visible only in a distinct area of the sections just beneath the long trichoid sensilla, which is the typical expression pattern characteristic of pheromone receptors. In this regard, the expression pattern of PxylOR4 is the same as PxylOR1, as we expected.

### Pheromone Binding Proteins (PBPs) could Increase the Response Sensitivity of Pheromone Receptors (PRs)

**Figure 6:**
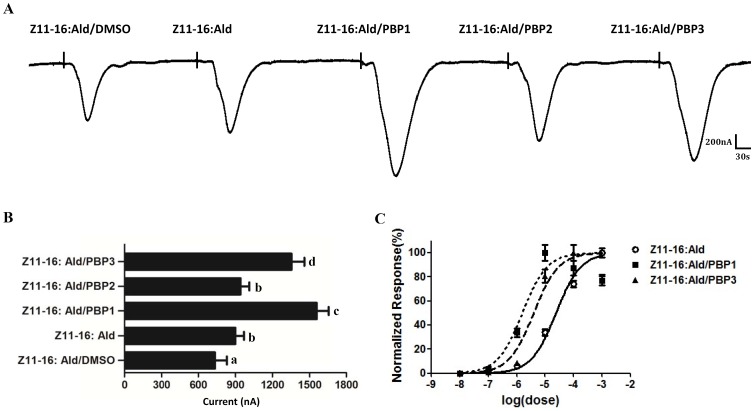
PBP-mediated responses of PxylOR1/PxylOrco *Xenopus* oocytes. (**A**) Inward current responses of PxylOR1/PxylOrco *Xenopus* oocytes in response to 10^−5^ M of Z11-16: Ald solubilized by DMSO, 1XRinger, or each of three PxylPBPs, respectively. (**B**) Response profile of PxylOR1/PxylOrco *Xenopus* oocytes. Error bars indicate SEM (*n = *5). Statistical comparison of responses of oocytes was assessed using one-way analysis of variance (ANOVA). (**C**) Dose–response profile of PxylOR1/PxylOR2*Xenopus* oocytes upon stimulation with different Z11-16: Ald concentrations solubilized by DMSO (n = 5), 1 µM PxylPBP1 (n = 4) and 1 µM PxylPBP3 (n = 4), respectively. Responses are normalized by defining the maximal response as 100%. Error bar indicates SEM.

Initially, we assessed all three PxylPBP types for their capability to replace the organic solvent DMSO in the functional electrophysiological recordings of PxylOR1/PxylOrco expressing oocytes. We compared the receptor responses to Z11-16: Ald solubilized by DMSO, 1XRinger, or each of three PxylPBPs. Z11-16: Ald (10 µM) dissolved in DMSO elicited much weaker receptor response than that solubilized by each of three PxylPBPs and slightly weaker than that dissolved in 1XRinger (Figure 6A, B). This result is qualitatively similar to previous report on heterologous expression of PRs in HEK293 cells [24].

**Figure 7:**
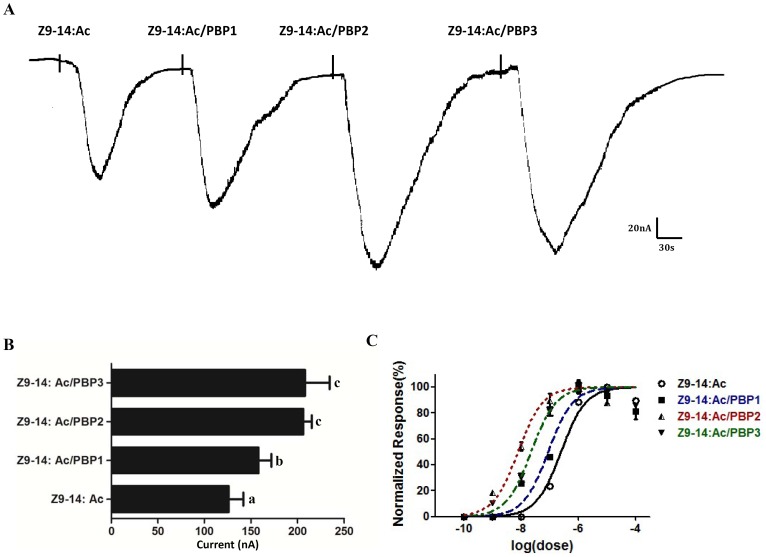
PBP-mediated responses of PxylOR4/PxylOrco *Xenopus* oocytes to Z9-14: Ac. (**A**) Inward current responses of PxylOR4/PxylOrco *Xenopus* oocytes in response to 10^−5^ M of Z9-14: Ac solubilized by 1XRinger, or each of three PxylPBPs, respectively. (**B**) Response profile of PxylOR4/PxylOrco *Xenopus* oocytes. Error bars indicate SEM (*n = *5). Statistical comparison of responses of oocytes was assessed using one-way analysis of variance (ANOVA). (**C**) Dose–response profile of PxylOR4/PxylOrco *Xenopus* oocytes upon stimulation with different Z9-14: Ac concentrations solubilized by 1XRinger (n = 7), 1 µM PxylPBP1 (n = 4), 1 µM PxylPBP2 (n = 4), 1 µM PxylPBP3 (n = 4), respectively. Responses are normalized by defining the maximal response as 100%. Error bar indicates SEM.

Next, we assessed all three PxylPBP types for their physiological sensitivity to pheromone ligands. We first compared the receptor responses to pheromone ligands solubilized by 1XRinger, or each of three PxylPBPs. For PxylOR1, Z11-16: Ald (10 µM) dissolved in PxylPBP1 and PxylPBP3 elicited a much more robust response than that dissolved in 1XRinger (Figure 6A, B). For PxylOR4, Z9-14: Ac (10 µM) dissolved in all three PxylPBPs elicited a more robust response than that dissolved in 1XRinger (Figure 7A, B). We then assessed if the PxylPBPs could increase physiological sensitivity to corresponding pheromone ligands in a dose-dependent fashion. The results show that all EC50s decrease to a certain extent. For PxylOR1, the sensitivity of the PxylOR1/PxylOrco expressing cells to Z11-16: Ald is one order of magnitude higher in the presence of PxylPBP3 than in the presence of 1XRinger, while the sensitivity is about two orders of magnitude higher in the presence of PxylPBP1 than in the presence of 1XRinger (Figure 6C). For PxylOR4, the sensitivity of the PxylOR4/PxylOrco expressing cells to Z9-14: Ac is about one or two orders of magnitude higher in the presence of all three PxylPBPs than it is in the presence of 1XRinger (Figure 7C).

**Figure 8:**
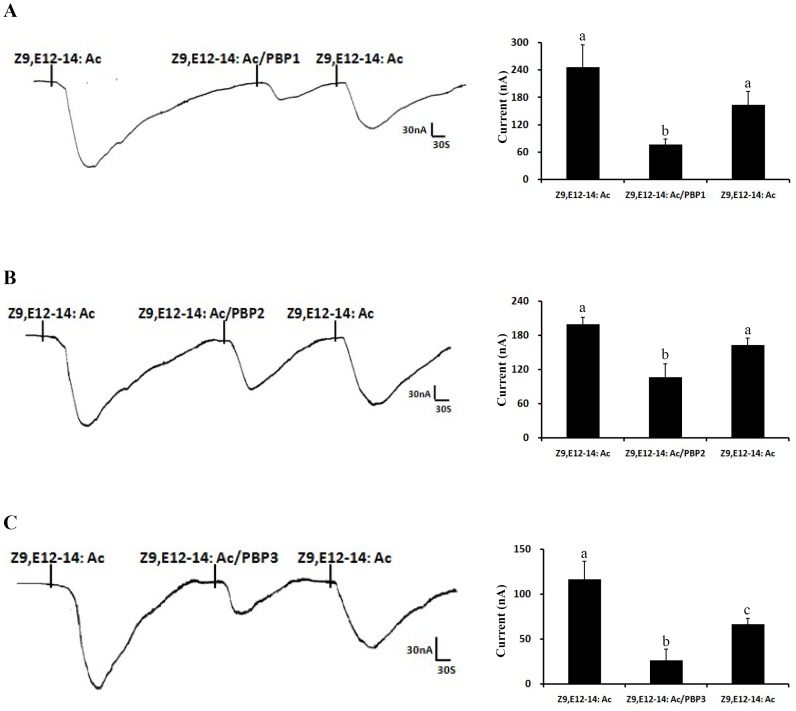
PxylOR4/PxylOrco *Xenopus* oocytes to Z9, E12-14: Ac of PxylPBP1-mediated (A), PxylPBP2-mediated (B), PxylPBP3-mediated (C)response, respectively. (Left)Inward current responses of PxylOR4/PxylOrco *Xenopus* oocytes in response to 10^−5^ M of Z9, E12-14: Ac solubilized by 1XRinger, PxylPBP and 1XRinger successively. (Right) Response profile of PxylOR4/PxylOrco *Xenopus* oocytes. Error bars indicate SEM (*n = *3). Statistical comparison of responses of oocytes was assessed using one-way analysis of variance (ANOVA).

We next studied further the influence of all three PxylPBP genes on the analog Z9, E12-14: Ac in the functional electrophysiological recordings of PxylOR4/PxylOrco expressing oocytes. We compared the receptor responses to Z9, E12-14: Ac solubilized by 1XRinger, or each of the three PxylPBPs. Interestingly, Z9, E12-14: Ac (10 µM) dissolved in each of the three PxylPBPs elicited significantly weaker response than that solubilized by 1XRinger (Figure 8). That is to say, the sensitivity of PxylOR4 to Z9, E12-14: Ac declines when adding PxylPBPs into the response system.

## Discussion

In the present study, we have identified six receptors from *P. xylostella* that could be assigned to the relatively conserved group of moth pheromone receptors in an insect OR phylogenetic tree (Figure 2). The predominant expression of these six receptors in male antennae in the gene expression profile experiments further supported the notion that they may represent receptors for pheromones (Figure 3). In addition to male-biased expression of all six receptors, PxylOR1 and PxylOR6 were faintly expressed in labial palps in male moths and PxylOR6 was also very faintly expressed in proboscises (Figure 2A). Given the fact that labial palps and proboscises are also olfactory appendages with chemosensory functions, the expression patterns of PxylOR1 and PxylOR6 genes could be understood. A previous report has also shown that moth pheromone receptor genes are expressed in secondary olfactory appendages [19]. Interestingly for PxylOR1 and PxylOR5, very faint PCR bands were obtained with cDNA from male moth legs. Sun et.al reported that three PxylPBPs were expressed in male moth legs [35], and Sengul et.al also reported that an OBP gene was expressed in male fly legs [43]. So there may also be associated olfactory function in male insect legs. Alternatively, the OBPs and/or PBPs may display biological functionality in a non-olfactory context.

In the *Xenopus*-based functional studies in the present study, PxylOR1 is the receptor for the main compound in the *P. xylostella* pheromone blend (*Z*11-16: Ald), which is consistent with the results of a previous study [20], providing verification of the reliability and stability of our experimental system. PxylOR4 was particularly interesting as it robustly responded to pheromone component, Z9-14: Ac as well as analog, Z9, E12-14: Ac. However, PxylOR4 is tuned to pheromone component Z9-14: Ac more sensitively. Previous research on electroantennogram recordings of male moth antennae indicates that Z9, E12-14: Ac could not elicit electrophysiological responses similar to Z9-14: Ac [35].

By comparing expression patterns between PxylOR1 and PxylOR4 in *in situ* hybridization experiments, the observation that the novel receptor type PxylOR4 was expressed in cells that could be confined to long sensilla trichodea further supports PxylOR4 as a pheromone receptor. This conclusion is different to a previous study [20]. Mitsuno et al. eliminated PxylOR4 as a candidate sex-pheromone receptor gene since no clear signal of the transcript was detected surrounded by a support cell expressing PxylPBP1 in olfactory sensilla. Based on the fact that there are more than one pheromone binding protein in the moth, we infer PxylOR4 as a pheromone receptor because it responsed to one pheromone component in the *Xenopus*-based functional studies. At the same time, Dr. Mitsuno did not mentioned Z9-14: Ac as one pheromone component possibly because different geographic populations of the moths have various pheromone components.

So far, we have identified the receptors to two pheromone components in *P. xylostella.* One is the first main component, Z11-16: Ald, and the other is one minor component, Z9-14: Ac. Pheromone receptors for two other common identified pheromone components - Z11-16: Ac and Z11-16: OH have not been determined. Here we could not identify the function of the other four cloned receptors. This may be due to the following reasons: a) these receptors could not be expressed in the *Xenopus* oocytes; b) the identification of the function of these four receptors may need other co-factors such as SNMPs in the system; c) there may be other yet unidentified ligands that function in male moth detection of conspecific females; d) these receptors do not respond to pheromone from P. xylostella but may respond to pheromones of closely related species.

In the *Xenopus*-based functional study three pheromone binding proteins (PxylPBP1-3) of the diamondback moth *P. xylostella* were added and assessed for their potential to increase the sensitivity of the identified pheromone receptors, PxylOR1 and PxylOR4. For PxylOR1, PxylPBP1 and PxylPBP3 could increase the sensitivity of the receptor complex to Z11-16: Ald by about one or two orders of magnitude. This result is difference from previous study [27]. Xu et al reported that bombykol dissolved in DMSO elicited robust receptor response, but very weak response when solubilized by BmorPBP1 and they suggested that PBP/pheromone complexes are not necessary for activation of moth ORs according to this result. But our conclusions are not conflicting. They emphasized the non- necessity of the PBP/pheromone complex and we want to emphasize the influence of PBPs to the sensitivity of PRs. And for PxylOR4 in our experiment, all three PxylPBPs could increase the sensitivity of the receptor to pheromone component Z9-14: Ac but significantly decreased the responsiveness of the receptor to the pheromone analog Z9, E12-14: Ac, as compared to when DMSO is used as a solvent in the system of electrophysiological recordings. In a fluorescence displacement binding assay reported previously, each of the three PxylPBPs could bind Z9-14: Ac and Z9, E12-14: Ac with similar sensitivity [35]. But when the solvent is changed to each of the three PxylPBPs, the response of PxylOR4-PxylOrco expressing cells to Z9-14: Ac and Z9, E12-14: Ac is different. According to this result, we deduce two following reasons: a) after PxylPBPs binding analog and carrying it to sensory neuron membrane, PxylPBPs do not release analog completely like pheromone; b)specific activation of the pheromone receptor in olfactory receptor neurons requires correct conformation of the pheromone/PBP complex. If the chemical is not a pheromone component, but instead a pheromone analog with a similar structure, the complex would have a reduced ability to activate downstream pheromone receptors.

## Supporting Information

Material S1 **Accession numbers for amino acid sequences of ORs in phylogenetic analyses.**
(DOCX)

## References

[1] Regnier FE, Law JH (1968) Insect pheromones. Journal of Lipid Research 9: 541–551. 4882034

[2] Leal WS (2013) Odorant Reception in Insects: Roles of Receptors, Binding Proteins, and Degrading Enzymes. Annual Review of Entomology 58: 373–391. 10.1146/annurev-ento-120811-153635 23020622

[3] Smith DP (2007) Odor and pheromone detection in *Drosophila melanogaster* . Pflügers Archiv European Journal of Physiology 454: 749–758. 17205355 10.1007/s00424-006-0190-2

[4] Hildebrand JG (1995) Analysis of chemical signals by nervous systems. Proceedings of the National Academy of Sciences 92: 67–74. 10.1073/pnas.92.1.67 PMC42818 7816849

[5] Masson C, Mustaparta H (1990) Chemical information processing in the olfactory system of insects. Physiological Reviews 70: 199–245. 2404289 10.1152/physrev.1990.70.1.199

[6] Blomquist GJ, Vogt RG (2003) Insect pheromone biochemistry and molecular biology: The biosynthesis and detection of pheromones and plant volatiles: Academic press.

[7] Rutzler M, Zwiebel LJ (2005) Molecular biology of insect olfaction: recent progress and conceptual models. Journal of Comparative Physiology A, Neuroethology, Sensory, Neural, and Behavioral Physiology 191: 777–790. 10.1007/s00359-005-0044-y 16094545

[8] Sato K, Pellegrino M, Nakagawa T, Vosshall LB, Touhara K (2008) Insect olfactory receptors are heteromeric ligand-gated ion channels. Nature 452: 1002–1006. 18408712 10.1038/nature06850

[9] Wicher D, Schafer R, Bauernfeind R, Stensmyr MC, Heller R, et al (2008) *Drosophila* odorant receptors are both ligand-gated and cyclic-nucleotide-activated cation channels. Nature 452: 1007–1011. 18408711 10.1038/nature06861

[10] Ishida Y, Leal WS (2005) Rapid inactivation of a moth pheromone. Proc Natl Acad Sci U S A 102: 14075–14079. 16172410 10.1073/pnas.0505340102 PMC1216831

[11] Sakurai T, Mitsuno H, Haupt SS, Uchino K, Yokohari F, et al (2011) A single sex pheromone receptor determines chemical response specificity of sexual behavior in the silkmoth *Bombyx mori* . PLoS Genetics 7: e1002115. 21738481 10.1371/journal.pgen.1002115 PMC3128102

[12] Sakurai T, Nakagawa T, Mitsuno H, Mori H, Endo Y, et al (2004) Identification and functional characterization of a sex pheromone receptor in the silkmoth *Bombyx mori* . Proceedings of the National Academy of Sciences of the United States of America 101: 16653–16658. 15545611 10.1073/pnas.0407596101 PMC528734

[13] Nakagawa T, Sakurai T, Nishioka T, Touhara K (2005) Insect sex-pheromone signals mediated by specific combinations of olfactory receptors. Science 307: 1638–1642. 15692016 10.1126/science.1106267

[14] Krieger J, Grosse-Wilde E, Gohl T, Dewer YM, Raming K, et al (2004) Genes encoding candidate pheromone receptors in a moth (*Heliothis virescens*). Proceedings of the National Academy of Sciences of the United States of America 101: 11845–11850. 15289611 10.1073/pnas.0403052101 PMC511062

[15] Liu Y, Gu S, Zhang Y, Guo Y, Wang G (2012) Candidate Olfaction Genes Identified within the *Helicoverpa armigera* Antennal Transcriptome. PLoS ONE 7: e48260. 23110222 10.1371/journal.pone.0048260 PMC3482190

[16] Montagné N, Chertemps T, Brigaud I, François A, François MC, et al (2012) Functional characterization of a sex pheromone receptor in the pest moth *Spodoptera littoralis* by heterologous expression in Drosophila. European Journal of Neuroscience. 10.1111/j.1460-9568.2012.08183.x 22748123

[17] Wanner KW, Nichols AS, Allen JE, Bunger PL, Garczynski SF, et al (2010) Sex pheromone receptor specificity in the European corn borer moth, Ostrinia nubilalis. PLoS ONE 5: e8685. 20084285 10.1371/journal.pone.0008685 PMC2801615

[18] Grosse-Wilde E, Stieber R, Forstner M, Krieger J, Wicher D, et al (2010) Sex-Specific Odorant Receptors of the Tobacco Hornworm *Manduca Sexta*. Front Cell Neurosci 4. 10.3389/fncel.2010.00022 PMC2922936 20725598

[19] Forstner M, Breer H, Krieger J (2009) A receptor and binding protein interplay in the detection of a distinct pheromone component in the silkmoth Antheraea polyphemus. International journal of biological sciences 5: 745. 20011135 10.7150/ijbs.5.745 PMC2793307

[20] Mitsuno H, Sakurai T, Murai M, Yasuda T, Kugimiya S, et al (2008) Identification of receptors of main sex-pheromone components of three Lepidopteran species. European Journal of Neuroscience 28: 893–902. 18691330 10.1111/j.1460-9568.2008.06429.x

[21] Jordan MD, Anderson A, Begum D, Carraher C, Authier A, et al (2009) Odorant receptors from the light brown apple moth (*Epiphyas postvittana*) recognize important volatile compounds produced by plants. Chemical Senses 34: 383–394. 19293399 10.1093/chemse/bjp010

[22] Vogt RG (2003) Biochemical diversity of odor detection: OBPs, ODEs and SNMPs. Insect Pheromone Biochemistry and Molecular Biology. 391–445.

[23] Grosse-Wilde E, Gohl T, Bouché E, Breer H, Krieger J (2007) Candidate pheromone receptors provide the basis for the response of distinct antennal neurons to pheromonal compounds. European Journal of Neuroscience 25: 2364–2373. 17445234 10.1111/j.1460-9568.2007.05512.x

[24] Grosse-Wilde E, Svatos A, Krieger J (2006) A pheromone-binding protein mediates the bombykol-induced activation of a pheromone receptor in vitro. Chemical Senses 31: 547–555. 16679489 10.1093/chemse/bjj059

[25] Syed Z, Ishida Y, Taylor K, Kimbrell DA, Leal WS (2006) Pheromone reception in fruit flies expressing a moth’s odorant receptor. Proc Natl Acad Sci U S A 103: 16538–16543. 17060610 10.1073/pnas.0607874103 PMC1621046

[26] Hallem EA, Ho MG, Carlson JR (2004) The molecular basis of odor coding in the *Drosophila* antenna. Cell 117: 965–979. 15210116 10.1016/j.cell.2004.05.012

[27] Xu P, Hooper AM, Pickett JA, Leal WS (2012) Specificity Determinants of the Silkworm Moth Sex Pheromone. PLoS ONE 7: e44190. 22957053 10.1371/journal.pone.0044190 PMC3434217

[28] Tamaki (1977) Z-11-hexadecenal and Z-11-hexadecenyl acteact :sex pheromone components of the diamonback moth (Lepideptera :Plutellidae). Appl Entomol Zool 12: 208–210.

[29] Ando T, Koshihara T, Yamada H, Vu MH, Takahashi N, et al (1979) Electroantennogram activities of sex pheromone analogues and their synergistic effect on field attraction in the diamondback moth. Applied entomology and zoology 14: 362–364.

[30] Koshihara T, Yamada H (1980) Attractant activity of the female sex pheromone of diamondback moth, *Plutella xylostella* (L.), and analogue. Japanese Journal of Applied Entomology and Zoology 24: 6–12.

[31] Zilahi-Balogh G, Angerilli N, Borden J, Meray M, Tulung M, et al (1995) Regional differences in pheromone responses of diamondback moth in Indonesia. International Journal of Pest Management 41: 201–204.

[32] Michereff MFF, Vilela EF, Michereff Filho M, Mafra-Neto A (2000) Synthetic sex pheromone use for field trapping of diamondback moth males. Pesquisa Agropecuária Brasileira 35: 1919–1926.

[33] Môttus E, Nômm V, Williams I, Liblikas I (1997) Optimization of pheromone dispensers for diamondback moth *Plutella xylostella* . Journal of Chemical Ecology 23: 2145–2159.

[34] Chisholm M, Steck W, Underhill E, Palaniswamy P (1983) Field trapping of diamondback moth *Plutella xylostella* using an improved four-component sex attractant blend. Journal of Chemical Ecology 9: 113–118. 24408624 10.1007/BF00987775

[35] Sun M, Liu Y, Wang G (2013) Expression patterns and binding properties of three pheromone binding proteins in the diamondback moth, *Plutella xyllotella* . Journal of Insect Physiology 59: 46–55. 23147025 10.1016/j.jinsphys.2012.10.020

[36] Bohbot J, Vogt RG (2005) Antennal expressed genes of the yellow fever mosquito (Aedes aegypti L.); characterization of odorant-binding protein 10 and takeout. Insect Biochemistry and Molecular Biology 35: 961–979. 15978998 10.1016/j.ibmb.2005.03.010

[37] Livak KJ, Schmittgen TD (2001) Analysis of relative gene expression data using real-time quantitative PCR and the 2-[Delta][Delta] CT method. Methods 25: 402–408. 11846609 10.1006/meth.2001.1262

[38] Lu T, Qiu YT, Wang G, Kwon JY, Rutzler M, et al (2007) Odor coding in the maxillary palp of the malaria vector mosquito *Anopheles gambiae* . Curr Biol 17: 1533–1544. 17764944 10.1016/j.cub.2007.07.062 PMC3113458

[39] Wang G, Carey AF, Carlson JR, Zwiebel LJ (2010) Molecular basis of odor coding in the malaria vector mosquito Anopheles gambiae. Proceedings of the National Academy of Sciences 107: 4418–4423. 10.1073/pnas.0913392107 PMC2840125 20160092

[40] Krieger J, Raming K, Dewer YM, Bette S, Conzelmann S, et al (2002) A divergent gene family encoding candidate olfactory receptors of the moth *Heliothis virescens* . Eur J Neurosci 16: 619–628. 12270037 10.1046/j.1460-9568.2002.02109.x

[41] Yang Y, Krieger J, Zhang L, Breer H (2012) The olfactory co-receptor Orco from the migratory locust (*Locusta migratoria*) and the desert locust (*Schistocerca gregaria*): identification and expression pattern. International journal of biological sciences 8: 159. 22211114 10.7150/ijbs.8.159 PMC3248701

[42] Clyne PJ, Warr CG, Freeman MR, Lessing D, Kim JH, et al (1999) A novel family of divergent seven-transmembrane proteins: Candidate odorant receptors in *Drosophila* . Neuron 22: 327–338. 10069338 10.1016/s0896-6273(00)81093-4

[43] Sengul M, Tu Z (2008) Characterization and expression of the odorant-binding protein 7 gene in *Anopheles stephensi* and comparative analysis among five mosquito species. Insect Molecular Biology 17: 631–645. 18811600 10.1111/j.1365-2583.2008.00837.x

