# Supplementary material for: Identification and Characterization of Pheromone Receptors and Interplay between Receptors and Pheromone Binding Proteins in the Diamondback Moth, Plutella xyllostella
Source: PLoS One. 2013 Apr 23;8(4):e62098. doi: 10.1371/journal.pone.0062098 (PMC3633919; doi:10.1371/journal.pone.0062098)
Supplement: Material S1 — Accession numbers for amino acid sequences of ORs in phylogenetic analyses. (DOCX) [file pone.0062098.s001.docx]

| **Protein name** | **Accession number** | **Protein name** | **Accession number** |
| --- | --- | --- | --- |
| HarmOR2 | ADQ13177 | BmorOR25 | BAH66322 |
| HarmOR6 | ([Liu et al., 2012](#_ENREF_18)) | BmorOR26 | BAH66323 |
| HarmOR11 | ACF32965 | BmorOR27 | BAH66324 |
| HarmOR13 | ACJ12370 | BmorOR28 | BAH66325 |
| HarmOR14 | ACF32964 | BmorOR29 | BAH66326 |
| HarmOR15 | ([Liu et al., 2012](#_ENREF_18)) | BmorOR30 | BAH66327 |
| HarmOR16 | ACS45306 | BmorOR32 | BAH66328 |
| HarmOR7p | ([Liu et al., 2012](#_ENREF_18)) | BmorOR33 | BAH66329 |
| HarmOR8p | ([Liu et al., 2012](#_ENREF_18)) | BmorOR34 | BAH66331 |
| HarmOR10p | ([Liu et al., 2012](#_ENREF_18)) | BmorOR35 | BAH66332 |
| HarmOR12 | ACF32963 | BmorOR36 | BAH66333 |
| HarmOR17 | ([Liu et al., 2012](#_ENREF_18)) | BmorOR37 | BAH66334 |
| HarmOR18 | ACC63239 | BmorOR38 | BAH66335 |
| HarmOR20 | ACC63240 | BmorOR39 | BAH66336 |
| HarmOR21p | ([Liu et al., 2012](#_ENREF_18)) | BmorOR40 | BAH66337 |
| HarmOR21-2p | ([Liu et al., 2012](#_ENREF_18)) | BmorOR41 | DAA05997 |
| HarmOR22p | ([Liu et al., 2012](#_ENREF_18)) | BmorOR42 | BAH66338 |
| HarmOR24p | ([Liu et al., 2012](#_ENREF_18)) | BmorOR44 | BAH66339 |
| HarmOR25p | ([Liu et al., 2012](#_ENREF_18)) | BmorOR45 | BAH66340 |
| HarmOR27 | ([Liu et al., 2012](#_ENREF_18)) | BmorOR46 | BAH66341 |
| HarmOR28p | ([Liu et al., 2012](#_ENREF_18)) | BmorOR47 | BAH66342 |
| HarmOR29 | ([Liu et al., 2012](#_ENREF_18)) | BmorOR49 | BAH66343 |
| HarmOR30p | ([Liu et al., 2012](#_ENREF_18)) | BmorOR50 | BAH66345 |
| HarmOR31p | ([Liu et al., 2012](#_ENREF_18)) | BmorOR51 | BAH66346 |
| HarmOR32p | ([Liu et al., 2012](#_ENREF_18)) | BmorOR53 | BAH66347 |
| HarmOR33p | ([Liu et al., 2012](#_ENREF_18)) | BmorOR54 | BAH66348 |
| HarmOR35p | ([Liu et al., 2012](#_ENREF_18)) | BmorOR55 | BAH66349 |
| HarmOR36p | ([Liu et al., 2012](#_ENREF_18)) | BmorOR56 | BAH66350 |
| HarmOR38p | ([Liu et al., 2012](#_ENREF_18)) | MsexOR1 | ACM18059 |
| HarmOR39p | ([Liu et al., 2012](#_ENREF_18)) | MsexOR4 | ADM32897 |
| HarmOR40p | ([Liu et al., 2012](#_ENREF_18)) | MsexOR5 | ADM32898 |
| HarmOR41p | ([Liu et al., 2012](#_ENREF_18)) | MsexOR6 | ([Grosse-Wilde et al., 2010](#_ENREF_24)) |
| HarmOR42p | ([Liu et al., 2012](#_ENREF_18)) | MsexOR7 | ([Grosse-Wilde et al., 2010](#_ENREF_24)) |
| HarmOR43 | ([Liu et al., 2012](#_ENREF_18)) | MsexOR8 | ([Grosse-Wilde et al., 2010](#_ENREF_24)) |
| HarmOR44 | ([Liu et al., 2012](#_ENREF_18)) | MsexOR9 | ([Grosse-Wilde et al., 2010](#_ENREF_24)) |
| HarmOR45 | ([Liu et al., 2012](#_ENREF_18)) | MsexOR10 | ([Grosse-Wilde et al., 2010](#_ENREF_24)) |
| HarmOR46p | ([Liu et al., 2012](#_ENREF_18)) | MsexOR11 | ([Grosse-Wilde et al., 2010](#_ENREF_24)) |
| HarmOR48p | ([Liu et al., 2012](#_ENREF_18)) | MsexOR12 | ([Grosse-Wilde et al., 2010](#_ENREF_24)) |
| HvirOR1 | CAD31850 | MsexOR13 | ([Grosse-Wilde et al., 2010](#_ENREF_24)) |
| HvirOR2 | CAD31851 | MsexOR14 | ([Grosse-Wilde et al., 2010](#_ENREF_24)) |
| HvirOR3 | CAD31852 | MsexOR15 | ([Grosse-Wilde et al., 2010](#_ENREF_24)) |
| HvirOR4 | CAD31946 | MsexOR16 | ([Grosse-Wilde et al., 2010](#_ENREF_24)) |
| HvirOR5 | CAD31947 | MsexOR17 | ([Grosse-Wilde et al., 2010](#_ENREF_24)) |
| HvirOR6 | CAD31948 | MsexOR18 | ([Grosse-Wilde et al., 2010](#_ENREF_24)) |
| HvirOR7 | CAD31853 | MsexOR19 | ([Grosse-Wilde et al., 2010](#_ENREF_24)) |
| HvirOR8 | CAD31949 | MsexOR20 | ([Grosse-Wilde et al., 2010](#_ENREF_24)) |
| HvirOR9 | CAD31950 | MsexOR21 | ([Grosse-Wilde et al., 2010](#_ENREF_24)) |
| HvirOR10 | CAG38111 | MsexOR22 | ([Grosse-Wilde et al., 2010](#_ENREF_24)) |
| HvirOR11 | CAG38112 | MsexOR23 | ([Grosse-Wilde et al., 2010](#_ENREF_24)) |
| BmorOR1 | BAD69584 | MsexOR24 | ([Grosse-Wilde et al., 2010](#_ENREF_24)) |
| BmorOR2 | BAD69585 | MsexOR25 | ([Grosse-Wilde et al., 2010](#_ENREF_24)) |
| BmorOR3 | BAD89567 | MsexOR26 | ([Grosse-Wilde et al., 2010](#_ENREF_24)) |
| BmorOR4 | BAD89568 | MsexOR27 | ([Grosse-Wilde et al., 2010](#_ENREF_24)) |
| BmorOR5 | BAD89569 | MsexOR28 | ([Grosse-Wilde et al., 2010](#_ENREF_24)) |
| BmorOR6 | BAD89570 | MsexOR29 | ([Grosse-Wilde et al., 2010](#_ENREF_24)) |
| BmorOR7 | NP_001106227 | MsexOR30 | ([Grosse-Wilde et al., 2010](#_ENREF_24)) |
| BmorOR8 | BAH66308 | MsexOR32 | ([Grosse-Wilde et al., 2010](#_ENREF_24)) |
| BmorOR9 | BAH66309 | MsexOR33 | ([Grosse-Wilde et al., 2010](#_ENREF_24)) |
| BmorOR10 | DAA05970 | MsexOR34 | ([Grosse-Wilde et al., 2010](#_ENREF_24)) |
| BmorOR11 | BAH66310 | MsexOR35 | ([Grosse-Wilde et al., 2010](#_ENREF_24)) |
| BmorOR12 | BAH66311 | MsexOR36 | ([Grosse-Wilde et al., 2010](#_ENREF_24)) |
| BmorOR13 | BAH66312 | MsexOR37 | ([Grosse-Wilde et al., 2010](#_ENREF_24)) |
| BmorOR14 | BAH66313 | MsexOR38 | ([Grosse-Wilde et al., 2010](#_ENREF_24)) |
| BmorOR15 | DAA05974 | MsexOR39 | ([Grosse-Wilde et al., 2010](#_ENREF_24)) |
| BmorOR16 | BAH66314 | MsexOR40 | ([Grosse-Wilde et al., 2010](#_ENREF_24)) |
| BmorOR17 | BAH66315 | MsexOR41 | ([Grosse-Wilde et al., 2010](#_ENREF_24)) |
| BmorOR18 | BAH66316 | MsexOR42 | ([Grosse-Wilde et al., 2010](#_ENREF_24)) |
| BmorOR19 | DAA05977 | MsexOR44 | ([Grosse-Wilde et al., 2010](#_ENREF_24)) |
| BmorOR20 | BAH66317 | MsexOR45 | ([Grosse-Wilde et al., 2010](#_ENREF_24)) |
| BmorOR21 | BAH66318 | MsexOR46 | ([Grosse-Wilde et al., 2010](#_ENREF_24)) |
| BmorOR22 | BAH66319 | MsexOR47 | ([Grosse-Wilde et al., 2010](#_ENREF_24)) |
| BmorOR23 | BAH66320 | MsexOR48 | ([Grosse-Wilde et al., 2010](#_ENREF_24)) |
| BmorOR24 | BAH66321 |  |  |
